# Towards an open, collaborative, reusable framework for sharing hands-on bioinformatics training workshops

**DOI:** 10.1093/bib/bbw013

**Published:** 2016-03-16

**Authors:** Nathan S Watson-Haigh, Jerico Revote, Radosław Suchecki, Sonika Tyagi, Susan M Corley, Catherine A Shang, Annette McGrath

**Affiliations:** Australian Centre for Plant Functional Genomics, University of Adelaide, Urrbrae SA, Australia

**Keywords:** bioinformatics; training*;* workshop; reusable; collaborative; open access

## Abstract

There is a clear demand for hands-on bioinformatics training. The development of bioinformatics workshop content is both time-consuming and expensive. Therefore, enabling trainers to develop bioinformatics workshops in a way that facilitates reuse is becoming increasingly important. The most widespread practice for sharing workshop content is through making PDF, PowerPoint and Word documents available online. While this effort is to be commended, such content is usually not so easy to reuse or repurpose and does not capture all the information required for a third party to rerun a workshop. We present an open, collaborative framework for developing and maintaining, reusable and shareable hands-on training workshop content.

## Introduction

Technological changes in molecular sequence data generation and acquisition has made DNA sequencing central to many areas of life science research. A large amount of sequence data can now be generated in a few days. There has been a paradigm shift from data generation to data analysis, which has led to a surge in demand [1] for researchers with the bioinformatics skills necessary to analyse large data sets. Empowering non-bioinformaticians, through hands-on training courses, to take ownership of the analysis of their own data will improve knowledge and understanding of bioinformatics approaches and help ease the skills shortage.

## The demand for bioinformatics training

Many community surveys have been conducted in recent years to gain greater insight into the demand for bioinformatics training. These surveys have either tried to ascertain where life scientists require support or more specifically focus on bioinformatics training needs.

The Global Organisation for Bioinformatics Learning Education and Training (GOBLET) conducted an international survey investigating bioinformatics training needs (https://www.surveymonkey.com/s/GOBLETsurvey). Of the 498 respondents, 54% indicated a desire for training in data analysis and statistics, while 17% indicated a training need for using and adapting software tools. There was a clear preference for training material to be delivered online or by stand-alone workshops [2].

The outcome of an European Molecular Biology Laboratory (EMBL) Australia Bioinformatics Resource survey conducted in 2013 found an overwhelming need for bioinformatics training [3]. Most respondents (75%) indicated that training would be ‘very useful’, while only 2% of respondents felt that training would be of no benefit to them.

The Commonwealth Scientific and Industrial Research Organisation (CSIRO) Bioinformatics Core conducted a staff survey in 2011 to gauge the level of demand, among bioscientists and bioinformaticians, for courses in a range of topics relevant to bioinformatics. All respondents indicated an interest in undertaking training in at least one topic, with next-generation sequencing (NGS) analysis and R programming being most popular.

To build critical mass and meet a diverse range of training needs, a training partnership was developed between the CSIRO Bioinformatics Core and Bioplatforms Australia (BPA), which draws on trainers from across both organizations and their respective networks [4]. To date, training courses and workshops run through this collaboration have been oversubscribed, indicating a clear demand for more. For instance, our Introduction to NGS hands-on workshop has received 1038 applications for 726 places across 19 workshops in 8 cities hosted at 11 venues over the past 3 years.

To meet the demand for training workshops and to sustainably scale our efforts, we are adding new volunteer trainers to our network and running more courses across the country. Consequently, information about our workshop content needs to be available centrally and to be structured so that it is self-�explanatory, reusable and open, with minimal overhead. To make this possible, we have defined a framework that ensures all the information required for running and teaching a workshop is adequately and consistently described, is easily maintained by a geographically distributed network of trainers, optimized for reuse and is openly accessible.

In this article we describe this framework and how others can adopt our approach to develop and deliver shared workshop content that can be easily reused and repurposed.

## A framework for implementation and reuse of workshops

Our framework describes the key components of a reusable hands-on workshop, uses plain text files to record these and a distributed version control system (git) to manage collaborative content development and long-term maintenance (Table 1 provides a glossary of terms associated with our framework). The framework does not aim to guide curriculum development itself or provide guidance on educational approaches or learning processes. For trainers it provides a framework in which content can be developed so that it will be more easily reused and repurposed. Our companion paper shows how the information captured by our framework can be used to deploy a workshop into a cloud-based training environment [5], collectively referred to as the Bioinformatics Training Platform (BTP).

**Table 1.** A glossary of terms

**Term**	**Definition**
Commit	Saving changes, to one or more files, in a git repository.
Deploy	Setting up a BTP workshop on a Linux machine.
Fork	Create a copy of a GitHub hosted git repository in which you have write access.
Git	Git is a distributed revision control system, allowing many people to work on a project.
GitHub	Web-based Git repository hosting service, which offers all of the distributed revision control and source code management functionality of Git.
LaTeX	High-quality typesetting system, which uses plain text.
GNU Make	Tool for controlling the generation of files from a source.
Pull request	Request for the changes you have made, to a forked GitHub repository, to be applied to that repository.
Submodule	Git submodules allow you to nest Git repositories as a subdirectory of another Git repository.
Travis CI	Open-source hosted, distributed continuous integration service used to run automate tasks when commits are made to a GitHub project.
YAML	YAML Ain't Markup Language, a human readable data serialization format used for defining data mappings.

Developing successful bioinformatics training workshops can be challenging [6], time-consuming and therefore expensive, making the reuse of content developed and shared by others an attractive prospect.

GOBLET has recently relaunched its training portal [7], which lists a wealth of training materials spanning many topics. While many trainers have contributed their materials (e.g. presentations in PDF, PowerPoint and Word format) for others to use, on their own, many of these files do not provide sufficient information to successfully rerun a workshop without significant effort. The sharing of these files is a welcome first step, but they often fall short of being truly reusable. Files shared this way are difficult to keep up-to-date by anyone other than the original author. As a result, material risks becoming static and less useful for others. If there are multiple collaborators developing training material together, it can also be difficult to simultaneously manage multiple contributions in a cohesive manner.

The establishment of a structured framework in which hands-on bioinformatics training workshops can be developed in a way that facilitates sharing and reuse is timely.

## Essential components of a reusable workshop

For a workshop to be fully reusable, adequate information about four key components of a workshop needs to be captured: (1) presentation files; (2) tutorial exercises; (3) data sets and (4) tools used during the tutorial exercises.

Presentation files are used to introduce topics and key concepts to trainees. Tutorial exercises form a key component of a hands-on workshop by providing the trainee with an opportunity to learn through worked examples. Key learning outcomes are specified up front, questions posed throughout to test the trainee’s level of comprehension and optional/additional exercises provided for those who progress rapidly. Data sets used throughout the tutorial exercises should be representative of real data sets but small enough in size to run in reasonable time on the compute infrastructure available. Similarly, tools are an integral part of a hands-on workshop, and information about the tools used in the tutorial exercises and the data sets that they will use is critical to make a training workshop reusable.

## The BTP framework

This framework evolved out of our previously published NGS workshop [4]. Having run this workshop a few times, it became clear we needed greater flexibility to easily reuse individual components of the content, e.g. RNA-Seq section only, to deliver shorter, more focussed workshops. The NGS workshop was refactored into self-contained, reusable workshop modules to enable greater flexibility of reuse. For each module to stand alone it requires all the information regarding its tools, data sets, tutorial exercises and presentation files to be recorded. All the BTP-related content is available under a creative commons licence from our BPA-CSIRO-Workshops GitHub account (https://github.com/BPA-CSIRO-Workshops). This provides us with a central, publicly accessible location for all our workshop materials while also providing a means to establish structured workflows for the development and maintenance of that content.

**Figure 1.**
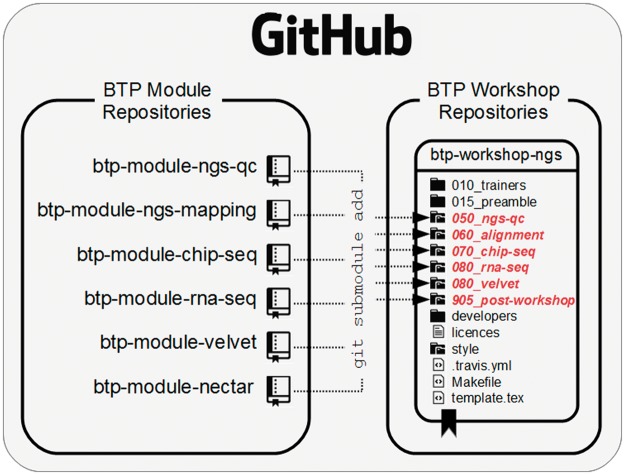
An overview of how the BTP module repositories relate to the BTP workshop repositories by way of git submodules.

The BTP framework contains two major components, the *module* and the *workshop* (Figure 1). Modules are the basic building blocks of workshop content. Each module and workshop is maintained as separate git repository on GitHub. We provide basic template repositories for the development of new modules (https://github.com/BPA-CSIRO-Workshops/btp-module-template) and workshops (https://github.com/BPA-CSIRO-Workshops/btp-workshop-template), enabling more rapid development and reuse of workshop content. Our framework represents a considerable effort to ease the burden on workshop content developers when attempting to write reusable workshop modules.

### BTP module template

The btp-module-template repository provides the directory structure, template files with inline documentation and README files to expedite the creation of new BTP workshop modules. A workshop module contains four subdirectories for storing files/information regarding the (1) data sets; (2) tools; (3) tutorial exercises and (4) presentations. The datasets and tools directories store information about where the data sets and tools for a given module can be obtained, how to install the tools and where on the filesystem data sets should reside. The presentations subdirectory is where the presentation files for the module are stored. The handout subdirectory contains a single LaTeX file together with any other files (e.g. .png) that are referred to in that LaTeX source. This file contains the tutorial exercises for the module and is compiled into a workshop handout document.

### BTP workshop template

The btp-workshop-template repository provides workshop developers with the components required for creating a new BTP workshop from pre-existing BTP modules. The workshop tutorial exercise handout is available as a high-level LaTeX file (template.tex), which sets out workshop-level information such as workshop title, date, venue and authors. By default, this LaTeX file also includes a Creative Commons Licence information page, a placeholder table of trainer details and information page on the structure/formatting of the handout. Our BTP LaTeX package is included in this template repository as a git submodule, thus providing access to the LaTeX styling information it defines [5].

A Makefile together with the template.tex file provides a simple way to build a trainer and trainee version of the workshop handout in PDF format. A simple ‘make tex_env’ uses information and scripts contained within the developers subdirectory to install a minimal TeX-Live environment. The execution of ‘make’ finds LaTeX files in the handout directories of any included workshop modules. The identified LaTeX files are included, in sorted order, into the template.tex file before trainer and trainee versions of the tutorial exercise handout is built.

In addition to these core components, the repository also includes files relating to more advanced features of the BTP framework, namely a .travis.yml file. The file contains instructions on the automated tasks to be performed by the Travis Continuous Integration (Travis-CI) service each time a commit is made to the repository. The use of Travis-CI (1) ensures LaTeX syntax errors are caught early; (2) helps during the process of merging a pull request by testing if the merge and PDF building can be completed without error; (3) ensures an up-to-date version of the workshop exercises PDF is always available online and ready for use as part of a workshop; (4) makes it unnecessary for every collaborator to set up his/her own local TeX environment.

## Using and reusing the BTP framework

The intention of our framework is to capture all the information required to make reusing hands-on workshop materials a painless reality. Our companion paper deals with aspects of using this framework to deploy workshops to (1) VirtualBox or VMware images, (2) the Australian NeCTAR Research Cloud and (3) Amazon Web Services (AWS) [5].

We present general workflows for the most common scenarios students and trainers will encounter while reusing existing BTP workshop content or using our framework to develop new content. Details regarding specific commands to be executed are provided in the README.md file of the btp-workshop-template repository (https://github.com/BPA-CSIRO-Workshops/btp-workshop-template).

## Workflow 1: Reusing an existing BTP workshop to run your own workshop

**Figure 2.**
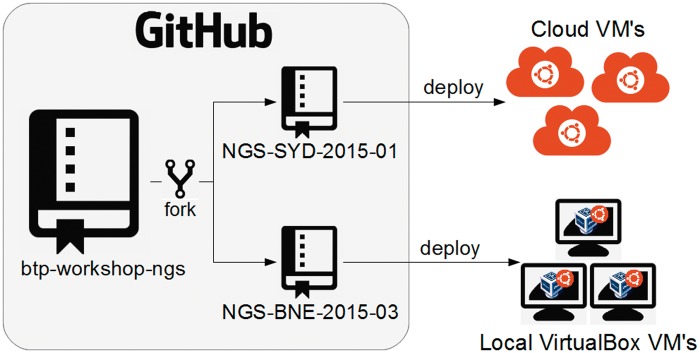
A schematic showing how a trainer would reuse the btp-workshop-ngs repository for delivering two of their own hands-on training workshops.

A trainer may want to reuse an existing BTP workshop, in its entirety, to run his/her own hands-on workshop. For instance, a trainer would like to deliver the BTP NGS workshop in January 2015 in Sydney and then in March 2015 in Brisbane (Figure 2). To do so, the trainer would fork the btp-workshop-ngs repository twice, renaming the forks NGS-SYD-2015-01 and NGS-BNE-2015-03, respectively. The two forks can then be customized by modifying the workshop venue, date, author(s), trainer details and content to satisfy the local needs of a particular workshop. Each of the two forks is then used to deploy virtual machines (VMs) for use in the hands-on training workshops. These forks act as a permanent record of what workshops were run and when. They also provide trainees with a point of reference to come back to the content at a later date if desired.

## Workflow 2: Updating BTP modules and workshops

**Figure 3.**
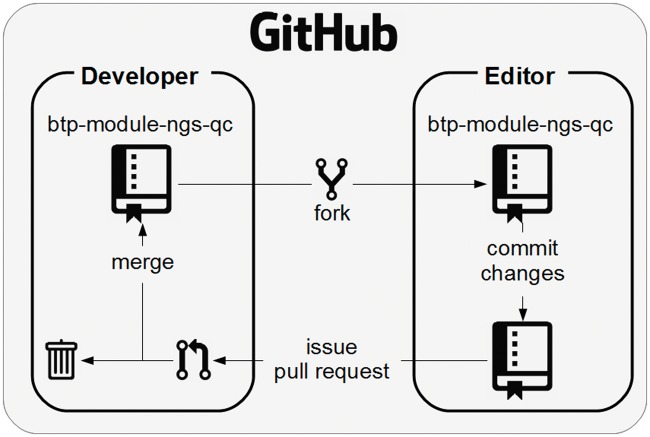
A schematic showing how contributions to a BTP module are made and incorporated into a master workshop module repository.

As the BTP modules and workshops mature and are reused by others, it is important to have a process in place whereby new contributions from others can be received, reviewed and included into the repository in a controlled manner. We use the git ‘fork & pull’ model, as it easily allows many people to contribute, while still having a mechanism in place that ultimately decides whether contributions are incorporated (Figure 3). To achieve this, a minimal set of users should be provided ‘write’ access to the master workshop and module repositories. These users are then responsible for ensuring the content is being developed in the intended direction. All contributions, including those from trainers who regularly deliver the content, must follow the fork & pull model to have changes incorporated into a repository.

BTP workshops repositories use git submodules for adding BTP workshop modules. Submodules are like links pointing to a specific revision of a repository (Figure 1). This ensures that changes to submodules do not automatically propagate into any repositories that include them. Therefore, to propagate changes in module content to a workshop, it is necessary to explicitly ask git to update the submodules.

## Workflow 3: Developing your own BTP module

Developing a workshop module using the BTP framework allows others to more easily reuse the content, as it can be easily incorporated into a BTP workshop by following workflow 4. The btp-module-template repository is used as the starting point for developing a new BTP module. It provides the structure, template files and documentation necessary to develop a new BTP module.

### Presentations

Presentations developed for introducing and exploring core concepts covered by a module are to be placed in the presentations subdirectory. We make no specific recommendations on file formats.

### Specifying the tools

**Figure 4.**
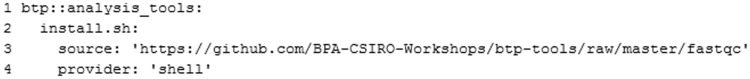
An example YAML-formatted file showing how a tool required by a BTP module is specified. Lines 2–3 describe where the tool is to be obtained from, while line 4 indicates what type of file is specified so a deployment script can deal with it appropriately. Lines 2–4 are repeated for each tool the workshop requires.

The tools used in the module’s tutorial exercises are specified using a YAML formatted file (tools/tools.yaml). A tool is defined by specifying a URL to an installation script (Figure 4) or Debian package. This information can then be used during the deployment of a workshop for setting up and configure only the tools required for the module. We maintain a central repository (btp-tools) of scripts for all the tools currently used by our BTP modules, which module developers can use.

**Figure 5.**
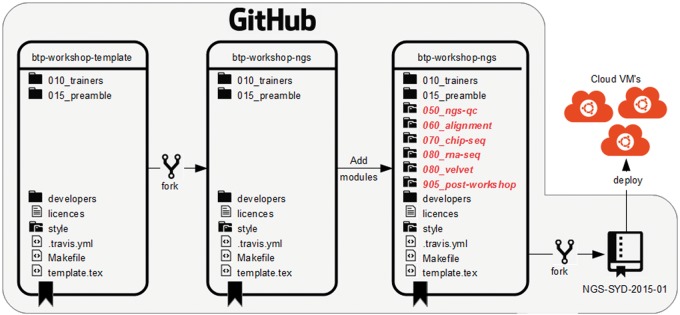
A schematic showing how the btp-workshop-ngs repository could be created using our framework.

### Specifying the data sets

Similarly, the data sets used by a module’s tutorial exercises are specified in a YAML formatted file (datasets/data.yaml). The file contains two key pieces of information: (1) where to obtain the data set files and (2) where the data set should be located on the filesystem following the deployment of a workshop. The locations should be in agreement with what is specified in the tutorial exercises for the module. It is useful to link to copies of data sets maintained on local, fast, highly available storage so as to improve performance when potentially large data sets are being downloaded. For instance, we maintain publicly accessible copies on both the NeCTAR Research Cloud and the Amazon Cloud.

### Writing tutorial exercises

The BTP framework uses the plain text document preparation system, LaTeX, for writing tutorial exercises. There are several reasons why we chose LaTeX rather than a more familiar software to prepare the handout exercises. First, writing documentation that contains code, scripts and commands for execution by a user is challenging in a word processor like Microsoft Word, particularly if the documentation is also to be used electronically and commands are to be copied and pasted from it. For instance, characters used commonly in code and at the command line (e.g. quotes, hyphens, double-hyphens and tilde) can be ‘auto-corrected’ by MS Word, thus causing problems when copying and pasting commands. Secondly, LaTeX is a plain text format which is easily version controlled and does not require any special software for editing. Thirdly, LaTeX allows multiple different outputs to be generated from a single source. For example, a trainer’s handout and a trainee’s handout can be generated from the same source files but differ from each other in terms of content and styling. None of these are easily achieved using Microsoft Word. We addressed these issues with the development of our BTP LaTeX package [5], which has now been refactored into its own repository (btp-handout-style) to enable easier reuse. This package is included in the btp-workshop-template repository as a git submodule. Therefore, tutorial exercises written for a BTP module will have immediate access to the LaTeX environments it defines.

Module tutorial exercises are written in a single LaTeX file under the ./handout directory. The btp-module-template provides a template file (./handout/example.tex) from which to start. It includes detailed help as LaTeX comments and example content, all specifically aimed at those not familiar with writing LaTeX. A module’s title, authors and contributors are specified by using \setModuleTitle{}, \setModuleAuthors{} and \setModuleContributions{}, respectively. These are used in rendering the cover page of a module.

Additional LaTeX knowledge will be required if the content developer wants to use lists, figures or tables. The ./handout/example.tex file provides examples for each of these and provides links to online tools such as Tables Generator (http://www.tablesgenerator.com/latex_tables) to help those less familiar with LaTeX.

## Workflow 4: Developing your own BTP workshop

The btp-workshop-template repository is the starting point for putting together a new BTP workshop. Briefly, the btp-workshop-template repository is forked to provide a starting point for the new master BTP workshop. Existing BTP modules are then added, using git submodules, to the newly created master BTP workshop repository. The submodule directory name should contain a three-digit prefix to indicate the order in which the modules are run by the workshop. Finishing touches are applied by updating the list of trainers that will deliver the content of the new workshop. Figure 5 shows an overview of this process. Workflow 1 can then be used when the time comes to run the hands-on workshop.

**Figure 6.**
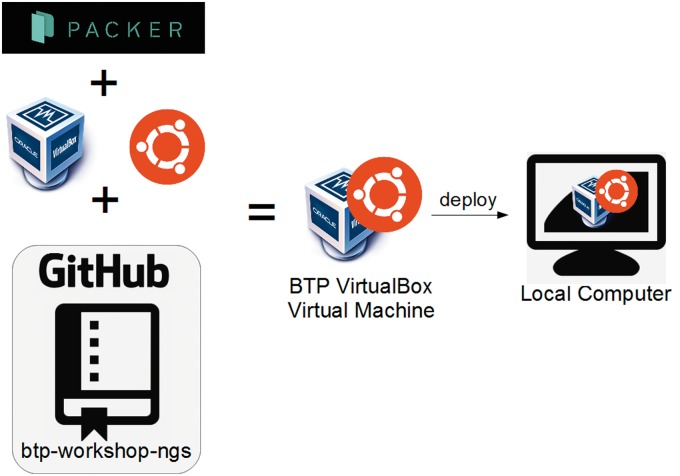
A schematic showing the process of building a VirtualBox image for the btp-workshop-ngs workshop repository.

## Workflow 5: Reusing an existing BTP workshop for self-directed learning

A self-directed learner might like to work through the contents of an existing BTP workshop in their own time and at their own pace. A VirtualBox image containing all the tools, data sets, tutorial exercises and presentation files defined by a BTP workshop can be built. The image can then be used by VirtualBox to create a Virtual Machine running on the learner’s local desktop computer. To create the VirtualBox image, (1) install Packer (https://www.packer.io/) and VirtualBox (https://www.virtualbox.org/), (2) clone the required BTP Workshop repository from GitHub and (3) issue a Packer build process (Figure 6). The image can then be used to boot a new VirtualBox virtual machine, providing a user with a self-contained training platform. A similar process can be used to create an image for use with VMWare. Revote *et al.* (2016) provide pre-built virtual machine images for several BTP workshops for use with VirtualBox and VMWare.

## Discussion

Bioinformatics training workshops take considerable time to develop and rely on experienced bioinformaticians to provide content and context. Hence, existing workshops are extremely valuable training resources, and the training community would benefit greatly if they could be shared and reused more easily across institutional and country boundaries.

Software Carpentry (SWC) teaches researchers basic software skills and run hundreds of workshops around the world every year and similarly operate with a geographically dispersed network of trainers. They also develop and maintain open access teaching material on GitHub to facilitate content reuse. However, as students are required to bring their own devices, SWC does not attempt to provide a consistent software training environment and thus does not attempt to capture the same information as we do in our framework. Flexibility in workshop delivery is afforded by the fact that they are training students to program using a text editor and the Unix environment rather than using specific data sets and running specific tools on those data sets to demonstrate key learning objectives. In this article we have defined a simple framework to enable workshops to be managed, developed collaboratively and shared with the broader community for reusing and repurposing.

### Open and collaborative framework

Our framework provides an open and consistent way to describe the tools, data sets, tutorial exercises and presentation material that together comprise all of the necessary elements of a bioinformatics training workshop. Before the development of this framework, we relied on a time-consuming manual process to manage, collate and distribute information about these elements to our trainer network. As individuals in the network developed content independently, many people had fragments of information pertaining to the workshop, all of which were essential to run a workshop. This framework centralizes the necessary information about a workshop and structures it in a sensible, consistent and transparent way that is readily understood.

The framework has provided an easy way to share information about content updates. Often the trainer who will deliver the material is not the one who developed it, and as we operate as a volunteer network, it is important that trainers can readily and quickly see changes to the content they will deliver. GitHub’s features enable trainers to quickly update their own content and for other trainers to easily see what content had changed.

The framework allows us to share our novel approach to fully describing a training workshop with others in the international bioinformatics community. By making the material open, transparent and accessible to all, we maximize the reuse potential of our material. Open access also increases the opportunities for driving further development by allowing others to build and extend on our work.

A collaborative contribution may be a major addition to a module, an entirely new module or a new workshop that comprises a different combination of existing or new BTP modules. If a collaborative contribution is a sensible addition to a module or workshop, the content can be merged into the master BTP workshop repository and can then be used and reused for future workshops.

Using this open and collaborative framework, our distributed trainer network has been able to (1) maintain the shared content to keep it current, (2) update tools and data sets, (3) update and fix errors that might arise in the training manual text over time (e.g. changed web service URLs) and (4) manage the merging of content edits into our existing workshop modules. This framework enables the repository to be a dynamic resource with up-to-date content.

### Modular and reusable

Our original workshop content [4] was stored as a single monolithic git repository, which contained tutorial exercises, shell scripts for the set-up of tools and data sets and a LaTeX style package, initially, for a 3-day Introduction to NGS course. While this made the workshop easy to rerun in its entirety, it was not flexible enough to encourage reuse of individual parts of the workshop or provide a framework for developing new content. The modularization of our workshops now offers considerable flexibility in reusing material and greater ease in maintaining content. Minor edits to specific components of the content, e.g. RNA-Seq, can be made to a module without having to edit the entire workshop content.

We provide templates and documentation to facilitate those who want to mix-and-match existing modules to develop a new workshop or those who want to develop entirely new modules for the community. It is now possible to create and run bespoke workshops of various lengths to different audiences and to respond quickly to community bioinformatics training needs. For instance, we have created and run 1-day Introductory RNA-Seq workshops comprising the QC, alignment and RNA-Seq modules only. Furthermore, we have developed and run half-day workshops on introductory NGS data analysis for the EMBL Australia PhD programme annually. This workshop comprises the QC and alignment modules only along with introductory lectures on NGS and its applications. Previously, workshop content that was not to be used in a shorter course had to be deleted from the workshop tutorial exercises and the associated tools and data sets deleted from the workshop image to be used in the cloud. Consequently, this represents a significant step forward for flexibility and reusability.

The modular nature of our framework means there is a concise, self-contained description of the workshop requirements in terms of the tools, data sets and tutorial exercises. This means we only need to maintain the workshop metadata.

Using our framework, we are continuing to develop new content with external collaborators. A ‘Cancer Genomics’ workshop with the European Bioinformatics Institute (EBI) and a ‘De Novo Genome Assembly’ workshop with The Genome Analysis Centre (TGAC) are currently being developed. The flexibility of our framework makes it possible for us to reuse material from our existing workshops, particularly the QC module. The new courses also provide an opportunity to develop new course-specific modules, which, like existing BTP modules, will be available for others to use within BTP workshops. Consequently, as the number of BTP modules grow, we gain greater flexibility to ‘plug and play’ individual modules in the creation of new workshops with different emphases, and to do so quickly.

### Best practice

Considering the time, effort and expertise invested into developing bioinformatics training workshops, it is important that others can easily cite this work. We advocate the use one of the many services (e.g. Australian National Data Service, Figshare, GitHub) that provide minting services to generate a DOI for online digital works as best practice. Online documentation outlining the procedure for generating a DOI for git repositories hosted on GitHub is available at https://guides.github.com/activities/citable-code/.

If others find benefits in our framework and begin to develop new modules and workshops, we will see a corresponding increase in the number of git repositories being hosted online. The distributed nature of git means that it may become difficult to search for and identify modules and workshops using the BTP framework. Therefore, looking to the future we would like to see a mechanism whereby BTP modules and workshops are easily discoverable so that they are even more easily reused and content development efforts are not needlessly duplicated.

**Key Points**

- A framework in which workshop content becomes truly reusable, not just available.
- A framework to facilitate open, collaborative content development and maintenance.
- A framework that can be readily adopted by the bioinformatics training community for contributing, developing and sharing workshop content.

## Funding

Bioplatforms Australia is funded by the Australian government through the Collaborative Research Infrastructure Scheme, the National Collaborative Research Infrastructure Strategy and the 2009 Super Science Initiative. This works was also supported by AWS with an Education Grant award. NeCTAR is an Australian Government project conducted as part of the Super Science initiative and financed by the Education Investment Fund. The CSIRO Bioinformatics Core is funded by CSIRO’s Transformational Biology Capability Platform. The Australian Centre for Plant Functional Genomics (ACPFG) is funded by the University of Adelaide, the South Australian Government and the Grains Research and Development Corporation (GRDC).

## Notes

**Nathan S. Watson-Haigh** is a bioinformatician with expertise and interest in wheat genomics and transcriptomics, systems biology approaches, de novo genome assembly, scientific computing infrastructure, cloud computing and workshop development and delivery.

## Notes

**Jerico Revote** is a software developer at the Monash eResearch Centre involved in research collaborations and R@CMon—the Research Cloud at Monash University.

## Notes

**Radoslaw Suchecki** is a bioinformatician with an interest in reproducible and scalable research, currently focusing on genomics and transcriptomics of bread wheat. He develops high-performance computational pipelines along with web applications for integration and visualization of biological data.

## Notes

**Sonika Tyagi** is a bioinformatician working in genomics whose main focus is on high-throughput sequence analysis. She is developing computational methods and protocols for transcriptomics, post-transcriptional gene regulation analysis, exome and variation analysis.

## Notes

**Susan Corley i**s a bioinformatician working at the NSW Systems Biology Initiative at UNSW. She is actively involved in several collaborations that use NGS data to investigate the cause and effect of mammalian tissue function and disease.

## Notes

**Catherine A. Shang** is a project manager at Bioplatforms Australia, working within large Australian collaborative systems biology research consortiums. Catherine is actively involved in establishing collaborative bioinformatics networks and developing and delivering open access bioinformatics training courses for the benefit of the Australian research community.

## Notes

**Annette McGrath** is the Bioinformatics Core Leader at the Commonwealth Scientific and Industrial Research Organisation. She leads a team of bioinformaticians involved in genomics research, bioinformatics infrastructure and tool development and with a strong interest in bioinformatics training and development.
